# Laryngeal manifestations in atypical recurrent aphthous stomatitis

**DOI:** 10.1016/S1808-8694(15)30514-0

**Published:** 2015-10-18

**Authors:** Ivan Dieb Miziara, Katia Cristina Costa, Ali Mahmoud, Raimar Weber, Niels Salles Willo Wilhelmsen, Rui Imamura

**Affiliations:** 1 Professor, São Paulo University Medical School. Head of the Stomatology Service, ENT Division, São Paulo University Hospital; 2 MD, Otorhinolaryngologist. Assisting Physician, Stomatology Service, ENT Division, São Paulo University Hospital; 3 MD, Otorhinolaryngologist. Assisting Physician, Stomatology Service, ENT Division, São Paulo University Hospital; 4 MD, Otorhinolaryngologist. Graduate Student, Otorhinolaryngology, São Paulo University Hospital; 5 Dental Surgeon, Graduate Student, Otorhinolaryngology, São Paulo University Hospital; 6 MD, Otorhinolaryngologist. Associate Physician, São Paulo University Hospital

**Keywords:** dapsone, aphthous stomatitis, larynx, fluorescence microscopy

## Abstract

Recurrent aphthoid stomatitis is characteristically observed in children and adolescents in the form of painful relapsing ulcers in the oral mucosa unaccompanied by evidences of systemic disease. The ulcers appear every one or two weeks for at least one entire year. Some patients suspected for recurrent aphthoid stomatitis develop lesions in atypical sites - mainly in the larynx - concurrently to the ones found in the oral mucosa.

**Aim:**

this study aims to describe a series of recurrent aphthoid stomatitis patients with atypical laryngeal injuries. Study design: this is a case series study.

**Materials and method:**

patients diagnosed with recurrent aphthoid stomatitis with oral mucosa ulcers and laryngeal symptoms without altered lab test results and no evidence of systemic disease underwent fibroscopic examination, oral and laryngeal biopsies, followed by specimen evaluation by direct immunofluorescence.

**Results:**

all six patients in this series had acute and chronic inflammatory processes according to pathology studies and negative direct immunofluorescence test results.

**Conclusion:**

laryngeal involvement in recurrent aphthoid stomatitis is rare. Therefore, during diagnostic examination thorough clinical history and meticulous physical examination accompanied by fibroscopic examination are necessary. When atypical lesions are found, biopsies for histological evaluation and direct immunofluorescence tests are required.

## INTRODUCTION

Recurrent aphthous stomatitis (RAS) is characteristically observed in children and adolescents in the form of painful relapsing ulcers in the oral mucosa unaccompanied by evidences of systemic disease. The ulcers appear every one or two weeks for at least one entire year^1^. Aphthous injuries apparently disconnected from underlying diseases or syndromes are somewhat frequently seen in our daily practice.

Oral injuries may mimic other conditions or be part of the clinical manifestations seen in various diseases, as is the case of autoimmune vesicular-bullous diseases. Fully evolved sores may look like pemphigus vulgaris (PV) or benign mucous membrane pemphigoid (BMMP), two conditions that frequently involve the laryngeal mucosa^2^, ^3^.

Cases suggestive of RAS with concurrent nonspecific laryngeal lesions ask not only for careful analysis of clinical history, physical examination, and lab workup, but may also require biopsy followed by pathology tests and immunofluorescence labeling so a proper diagnosis is established.

Direct immunofluorescence (DIF) on RAS, as described by Ship^4^, is utterly important in the establishment of a differential diagnosis from the atypical forms of RAS (in which test results are negative) and vesicular-bullous diseases (in which fluorescence is positive for oral and/or laryngeal epithelium)^4^.

## OBJECTIVE

This study aims to describe a series of six patients with oral ulcers and clinical history compatible with RAS, added by atypical laryngeal signs not compatible with any previously described manifestation of recurrent aphthous stomatitis.

## MATERIALS AND METHOD

This is a retrospective study featuring six consecutive patients seen from 2002 to 2004 by the stomatology group of a university hospital's ENT Clinic; all patients had aphthous injuries that appeared and evolved atypically.

Enrolled patients signed a free informed consent term. This study was approved by our institution's ethics committee under permit 876/04, as part of a Project investigating the clinical, pathological and genetic characteristics of RAS patients.

All enrolled patients were submitted to a protocol that consisted of general and clinical interviews, physical examination - mainly of the oral cavity, nasal fibroscopic examination using a flexible Olympus 3.4mm ENF Type P4 scope and a rigid 70-degree Storz (Hopkins) laryngeal scope connected to a 250-Watt halogen light source.

Lab workup included the following: complete blood count, coagulation profile, serum ferritin, G6PD dosage (glucose-6-phosphate dehydrogenase), antinuclear factor (ANF), rheumatoid factor (RF), serology for lues (RSS and FTA-ABS), anti-HIV 1 and 2, serum immunoglobulin (Ig) A, G and M, C-reactive protein.

Oral and laryngeal biopsies were performed to rule out other diseases affecting the oral cavity and to produce a differential diagnosis for RAS (Behçet disease, pemphigus, pemphigoid, erythema multiforme and others).

Oral biopsy procedure: patients were administered an injection of 2% xylocaine without vasoconstrictor; oral biopsy was performed using a 4-mm punch; specimens were obtained from the mucosa adjacent to the sore for direct immunofluorescence and from the transition zone between the injured and healthy portions of the mucosa for pathology workup.

Laryngeal mucosa biopsy was performed under general anesthesia. Patients underwent laryngeal micro surgery and had two specimens removed for pathology workup and DIF.

### Enrollment criteria:

• clinical history compatible with RAS, i.e., episodes of aphthous sores in the oral cavity occurring in monthly or shorter intervals for at least one year;
• presence of aphthous injury in the oral mucosa;
• absence of altered results in the tests order as part of the protocol;
• absence of clinical signs and/or test results compatible with systemic disease characterized by oral lesions.
• no use of topical corticosteroids on the sores at least two weeks prior to biopsy.

### Exclusion criteria:

• clinical history not compatible with RAS;
• presence of altered results in the tests order as part of the protocol;
• presence of clinical signs and/or test results compatible with systemic disease characterized by oral lesions.
• use of topical corticosteroids on the sores within two weeks prior to biopsy.

## RESULTS

All patients enrolled in our study had 'atypical aphthous injuries.' Three were males and three were females, with ages ranging between 22 and 64 years - mean age of 38.8 years (Table 1).

**Table 1:** Characteristics of patients with atypical RAS

Patient	Age	Gender	Complaints	Involved site	Lesion recurrence	Lesion size (diameter)
1	22	Female	Odynophagia + cough	Tongue Larynx (epiglottis)	Twice a month for 5 years	1,5 a 2,5 cm
2	26	Male	Odynophagia + Dysphagia + Hoarseness	Larynx (ventricular band) Healing tongue	Twice a month for 2 years	1,5 a 3,0 cm
3	29	Male	Odynophagia + Dysphagia + Hoarseness + Dyspnea	Larynx (epiglottis) Lips and tongue healing	Monthly for 18 months	1,5 a 3,0 cm
4	44	Female	Dysphagia + Dyspnea	Larynx (aryepiglottic fold) Lip	Every 20 days for 18 months	1,5 a 3,5 cm
5	48	Male	Dysphagia + Hoarseness	Larynx (ventricular band, arytenoids and epiglottis)	Monthly for 8 years	1,0 a 4,0 cm
6	64	Female	Odynophagia + Hoarseness	Larynx (arytenoids) Palate, tongue and lips healing	Monthly for 18 months	1,5 a 3,0 cm

**Figure 1:**
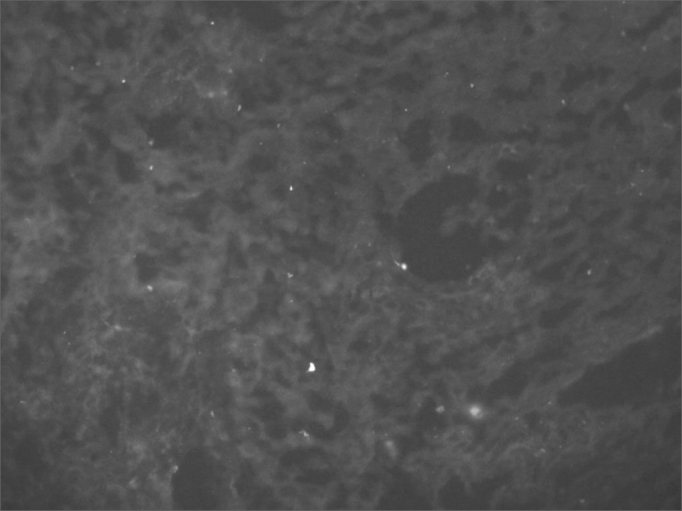
Direct immunofluorescence of oral mucosa: patients with atypical RAS - absent fluorescence.

Lab workup indicated the presence of oral and laryngeal mucosa ulcers and acute/chronic submucosal inflammation in all patients. Stains for fungi/granulomatosis were negative, as well as DIF (Fig. 1).

**Figure 2:**
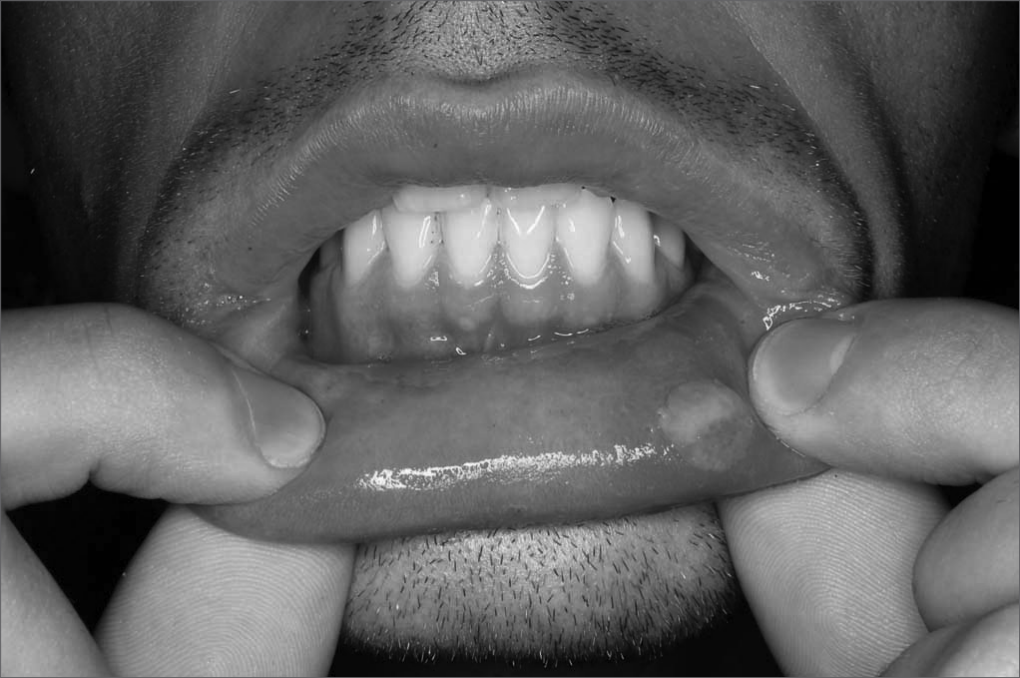
Aphthous lesion in oral mucosa of RAS patient.

Physical examination revealed four patients had oral lesions (Fig. 2), some active and some healing. Nasal fibroscopy showed additionally that all six patients had laryngeal injuries in various locations: epiglottis, arytenoids, and ventricular bands (Table 1).

The most frequently reported symptoms were pain, odynophagia, and dysphagia, all compatible with RAS. Hoarseness was also mentioned by 66.6% of the patients - all of whom found to have laryngeal injuries.

Most lesions were major, with sizes ranging between 1.0 cm (smallest) and 4.0 cm (largest).

Patients were treated with dapsone 100 mg/day initially combined with oral steroids - prednisone (30 mg/day); the latter was gradually removed after one month of treatment.

Patients evolved to full laryngeal injury remission and partial oral injury remission. They are currently being followed up on a monthly basis, and no adverse developments have been reported to date.

## DISCUSSION

Laryngeal injuries are not commonly seen in conjunction with RAS. We are not aware of any other study in which laryngeal involvement has been reported in cases of RAS. On the other hand, laryngeal involvement in cases of pemphigus vulgaris and other vesicular-bullous diseases is frequent^3^.

**Figure 3:**
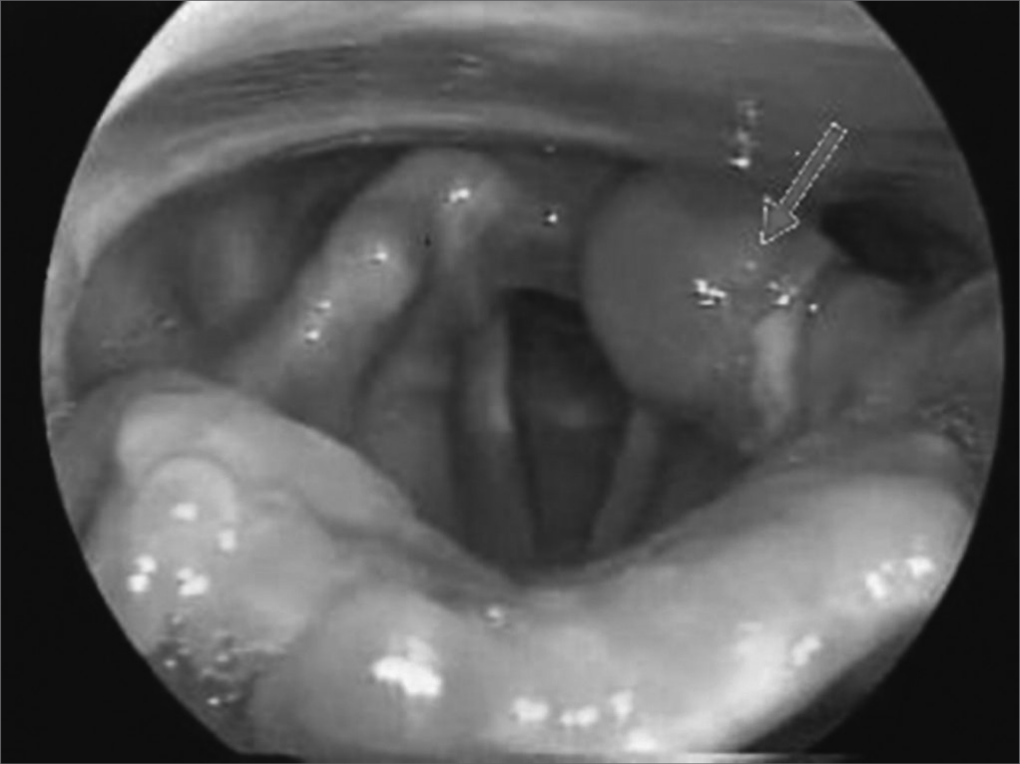
Atypical aphthous lesion in left arytenoidal region.

**Figure 4:**
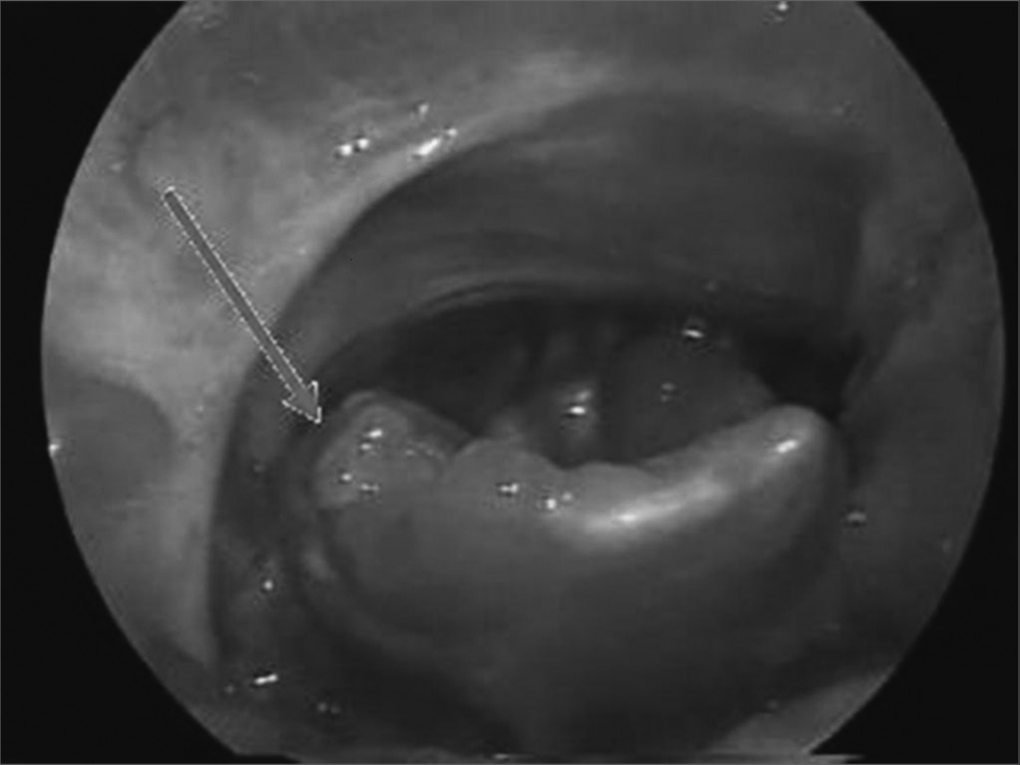
Atypical aphthous lesion in the lingual face of the right epiglottis.

Our study detected aphthous injuries concurrently to oral injuries in various sites in the larynx such as the epiglottis, aryepiglottic folds, ventricular bands, and arytenoids (Figure 3, Figure 4). Given such atypical manifestation, direct immunofluorescence was required to produce a differential diagnosis^4^. Direct immunofluorescence performed upon oral and laryngeal mucosa specimens yielded negative results.

Some authors have coined the term 'complex aphthosis' to characterize the constant presence of oral lesions or concurrent oral and genital lesions after ruling out Behçet disease (BD)^5^, ^6^, ^7^. In other words, complex aphthosis could also be an atypical manifestation of RAS. However, forty-two complex aphthosis patients studied by these authors failed to show laryngeal involvement^7^. And none of our patients had genital lesions and/or other signs and symptoms of BD.

The diagnosis of BD is eminently clinical, and requires the satisfaction of a number of conditions for confirmation: recurring oral aphthae, genital ulcers and/or uveitis and/or skin lesions^8^. Histological examination of the injuries produced by this disease shows vasculitis with perivascular leukocytic infiltration^9^. Once again, few are the cases of laryngeal involvement in BD patients. Such cases are usually reported when patients have advanced disease and complications such as pharyngolaryngeal stenosis^9^, ^10^, ^11^.

Although rare, laryngeal lesions occurring concurrently with oral lesions are observed in severe cases of erythema multiforme^12^. However, these ulcers are of acute origin and differ clinically from those of our patients, whose oral lesions are recurrent.

Laryngeal manifestations are also reported in the literature for Crohn's disease (CD)^13^, ^14^, ^15^. CD is a granulomatous inflammatory disease that may involve any portion of the gastrointestinal tract, including the oral cavity.

Most common symptoms are abdominal pain and diarrhea alternating to intestinal obstruction^16^. Skin, joints, and respiratory mucosa (nasal fossa, hypopharynx, and larynx) are cited as other possible areas of manifestation^17^.

The diagnosis for CD is produced from clinical and colonoscopy/biopsy findings positive for inflammatory granuloma. The most common finding in the CD involved larynx is edema, followed by ulcers and granulomatous processes^13^.

None of our patients had symptoms indicative of Crohn's or any other inflammatory intestinal disease throughout the follow-up period. Similarly, histology findings were never suggestive of Crohn's disease.

## CONCLUSION

Thorough clinical and physical examination, nasal fibroscopy included, are required during diagnosis, as laryngeal involvement is rare in recurrent aphthous stomatitis. When facing atypical lesions, biopsies are necessary for histology examination and direct immunofluorescence tests.
